# Serum 25-Hydroxyvitamin D Levels and Dry Eye Syndrome: Differential Effects of Vitamin D on Ocular Diseases

**DOI:** 10.1371/journal.pone.0149294

**Published:** 2016-02-19

**Authors:** Donghyun Jee, Seungbum Kang, Changzheng Yuan, Eunyoung Cho, Jorge G. Arroyo, The Epidemiologic Survey Committee of the Korean Ophthalmologic Society

**Affiliations:** 1 Department of Ophthalmology and Visual Science, College of Medicine, Catholic University of Korea, Suwon, Korea; 2 Department of Epidemiology and Biostatistics, Harvard T.H. Chan School of Public Health, Boston, Massachusetts, United States of America; 3 Department of Ophthalmology and Visual Science, Daejeon St. Mary’s Hospital, College of Medicine, Catholic University of Korea, Daejeon, Korea; 4 Department of Nutrition and Epidemiology, Harvard T.H. Chan School of Public Health, Boston, Massachusetts, United States of America; 5 Channing Division of Network Medicine, Brigham and Women's Hospital and Harvard Medical School, Boston, Massachusetts, United States of America; 6 Department of Dermatology, The Warren Alpert Medical School of Brown University, Providence, Rhode Island, United States of America; 7 Department of Ophthalmology, Beth Israel Deaconess Medical Center, Harvard Medical School, Boston, Massachusetts, United States of America; Boston University School of Medicine, UNITED STATES

## Abstract

**Purpose:**

To investigate associations between serum 25-hydroxyvitamin D levels and dry eye syndrome (DES), and to evaluate the differential effect of vitamin D on ocular diseases including age-related macular disease (AMD), diabetic retinopathy (DR), cataract, and DES.

**Methods:**

A total of 16,396 participants aged >19 years were randomly selected from the Korean National Health and Nutrition Examination Survey. All participants participated in standardized interviews, blood 25-hydroxyvitamin D level evaluations, and comprehensive ophthalmic examinations. DES was defined by a history of clinical diagnosis of dry eyes by a physician. The association between vitamin D and DES was compared to the associations between vitamin D and AMD, DR, cataract, and DES from our previous studies.

**Results:**

The odds of DES non-significantly decreased as the quintiles of serum 25-hydroxyvitamin D levels increased (quintile 5 versus 1, OR = 0.85, 95%CI: 0.55–1.30, P for trend = 0.076) after adjusting for potential confounders including age, sex, hypertension, diabetes, smoking status, and sunlight exposure times. The relative odds of DES (OR = 0.70, 95% CI: 0.30–1.64) and cataract (OR = 0.76, 95% CI: 0.59–0.99) were relatively high, while those of DR (OR = 0.37, 95% CI: 0.18–0.76) and late AMD (OR = 0.32, 95% CI: 0.12–0.81) were lower in men.

**Conclusions:**

The present study does not support an association between serum 25-hydroxyvitamin D levels and DES. The preventive effect of serum 25-hydroxyvitamin D may be more effective for DR and late AMD than it is for cataract and DES.

## Introduction

Dry eye syndrome (DES) is one of the most common ocular diseases and has been recognized as an important public health problem. Dry eye is associated with chronic eye pain and an increased risk of ocular surface diseases, such as corneal ulcers and corneal abrasions. DES is a multifactorial disorder of the tear film and ocular surface. During the past decades, many studies have demonstrated that inflammation is the core mechanism that plays a key role in the pathogenesis of DES [1]. Oxidative stress-induced inflammation may be involved in the functional decline of tear production [2, 3]. In addition, ocular surface inflammation was associated with excessive tear evaporation, which leads to tear film instability. We previously demonstrated that decreasing inflammatory cytokines and increasing anti-oxidant cytokines in tears can improve the symptoms and signs of DES [4, 5].

Vitamin D is a multifunctional hormone which plays a significant role in various biological functions in addition to its traditional role in regulating calcium homeostasis. Vitamin D can reduce inflammatory mediators and shows anti-oxidative functions [6–8]. Many human studies have shown an inverse relationship between vitamin D and chronic diseases associated with chronic inflammation including diabetes mellitus, hypertension, heart disease, multiple sclerosis, schizophrenia, and rheumatoid arthritis [9]. In the eye, vitamin D levels influence the development of a wide range of pathologies such as myopia [10], age-related macular degeneration (AMD) [11], diabetic retinopathy (DR) [12, 13], and uveitis. We previously reported an inverse association between vitamin D and AMD [14], DR [15], and age-related cataracts [16] in the Korean general population. However, to the best of our knowledge, no population-based epidemiologic studies have evaluated the associations between vitamin D levels and DES. The cornea, a main component of the ocular surface, contains vitamin D receptors and significant vitamin D concentrations, which suggests that vitamin D in the cornea may play a role in the anterior segment of the eye [17]. Therefore, we hypothesized that serum vitamin D levels play a role in DES development. To test our hypothesis, we examined the relationship between serum 25-hydroxyvitamin D levels and DES in a representative Korean population. In addition, we compared the association between vitamin D and DES to the associations between vitamin D and other ocular diseases including AMD, DR, and cataract from our previous reports to evaluate the differential effect of vitamin D on ocular diseases.

## Materials and Methods

### Study Population

**Fig 1:**
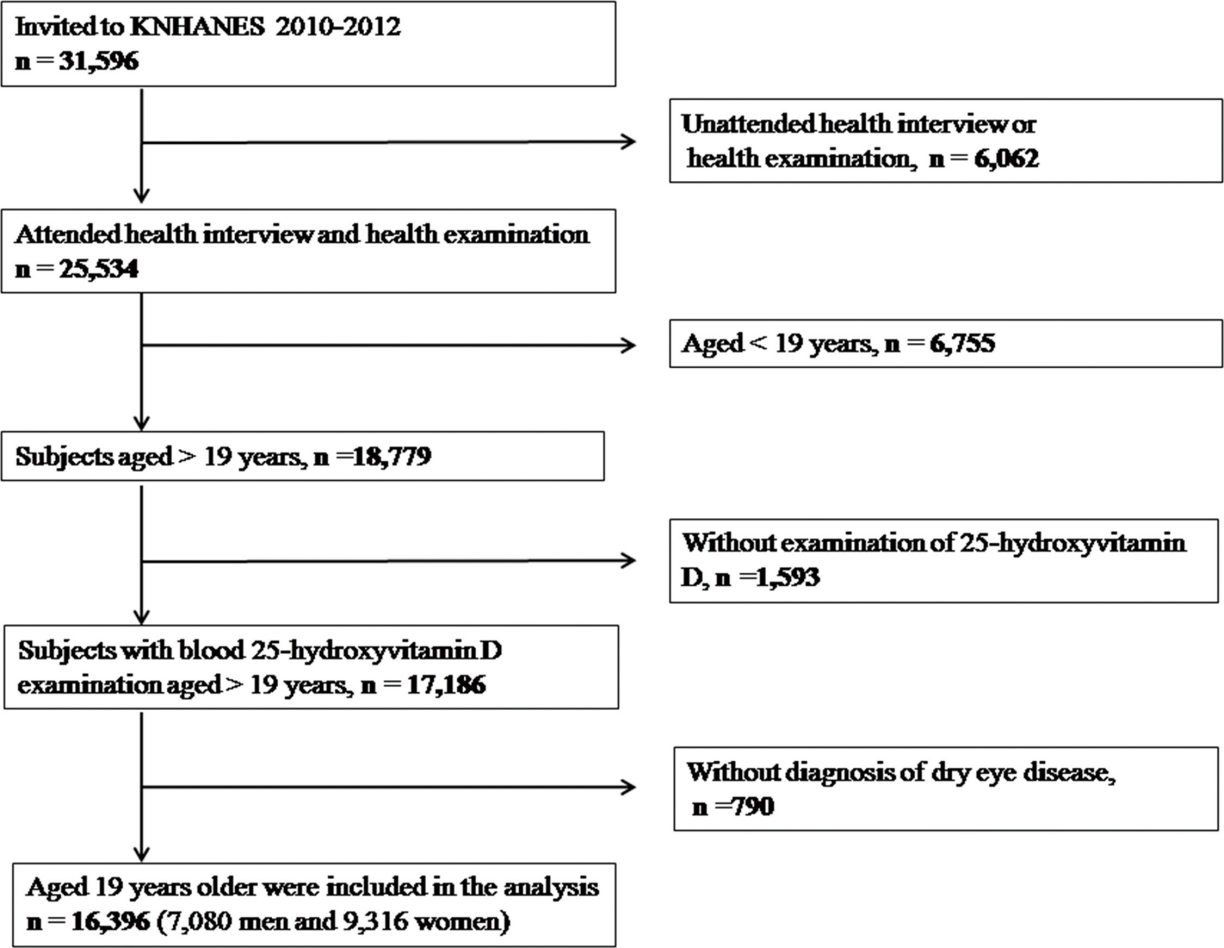
Flow diagram showing the selection of study participants.

This study used data acquired for the Korean National Health and Nutrition Examination Survey (KNHANES), which is a nationwide and population-based cross-sectional study conducted by the Korean Ministry of Health and Welfare and the Division of Chronic Disease Surveillance at the Korean Centers for Disease Control and Prevention. The study design and the methods on KNHANES are reported in detail elsewhere [18, 19]. KNHANES adopted a rolling sampling design, which was used to perform a stratified, complex, multistage probability cluster survey, with proportional allocations based on the National Census Registry for the non-institutional Korean civilian population. Data for the present study were obtained from the fifth (2010–2012) KNHANES to estimate the association between serum 25-hydroxyvitamin D levels and DES. 25,534 individuals who participated in KNHANES were enrolled. Of these, we excluded 6,755 subjects who were aged <19 years, 1,593 subjects who did not have their serum 25-hydroxyvitamin D levels measured, and 790 subjects who skipped the question on the diagnosis of DES. Finally, 16,396 participants aged ≥19 years were included (Fig 1). The study design followed the tenets of the Declaration of Helsinki for biomedical research. The institutional review board at the Catholic University of Korea in Seoul approved protocols for this study. All participants provided signed, written informed consent.

### Assessment of Serum 25-Hydroxyvitamin D Levels

The analysis of serum 25-hydroxyvitamin D levels is described previously [14–16]. Serum samples were collected after an 8-h fast, and serum 25-hydroxyvitamin D levels were measured by a radioimmunoassay kit (DiaSorin Inc., Stillwater, MN, USA) using a gamma counter (1470 Wizard, Perkin-Elmer, Waltham, MA, USA), which is often used in mass surveys such as KNHANES. KNHANES participates in the Vitamin D Standardization Program; hence, the measurement of 25-hydroxyvitamin D was standardized in accordance with the National Institute of Standards and Technology-Ghent University reference procedure [20]. All serum samples were appropriately processed, promptly refrigerated, and transported cold to a laboratory that is certified by the Korean Ministry of Health and Welfare at the Neodin Medical Institute in Seoul. Blood samples were analyzed within 24 hours after transportation. The detection limit for 25-hydroxyvitamin D by radioimmunoassay is 1.2 ng/ml, and the interassay coefficients of variation were 1.9–6.1% for samples taken between 2010 and 2012. In addition to analyzing serum 25-hydroxyvitamin D levels, fasting glucose, hemoglobin A1c (HbA1c), total cholesterol, and triglyceride levels were measured using a Hitachi 7600 clinical analyzer (Hitachi High-Technologies Corporation, Tokyo, Japan).

### Dry Eye Syndrome Assessments and Other Variables

DES examination and the diagnosis of KNHANES have been described in detail elsewhere [21, 22]. DES was defined by a history of a clinical diagnosis of dry eye syndrome by a physician, based on responses to a questionnaire. The questionnaire included the following yes/no question: "Have you ever been diagnosed by an ophthalmologist as having DES?" Demographic information was collected during health interviews. Height and weight measurements were obtained while subjects wore light clothing and no shoes. Body mass indices were calculated as follows: weight (kg)/height (m)^2^. Smoking status was self-reported, and subjects were classified as current smokers, past smokers, or non-smokers. Data on sunlight exposure were obtained by asking the participants whether they were exposed to the sun for < 2 h, 2–5 hrs, or ≥ 5 h per day. Three blood pressure measurements were taken at 5-min intervals using a sphygmomanometer with the subjects sitting down, and the average of the second and third measurements was used for the analysis. The presence of diabetes mellitus was defined as a fasting blood-glucose level ≥126 mg/dL or the use of anti-glycemic medication. The presence of hypertension was defined as a systolic blood pressure ≥140 mmHg, a diastolic blood pressure ≥90 mmHg, or use of antihypertensive medication.

### Statistical Analyses

Statistical analyses were performed using the IBM^®^ SPSS^®^ software version 18.0 (IBM, Armonk, NY, USA). Strata, sampling units, and sampling weights were used to obtain point estimates and standard errors. Participant characteristics were described using means and standard errors for continuous variables and percentages and standard errors for categorical variables based on the presence of DES. Analysis of variance or chi-square tests were used to compare the patients’ demographic characteristics. To evaluate the association between 25-hydroxyvitamin D and DES, serum 25-hydroxyvitamin D levels were categorized into quintiles, and simple and multiple logistic regression analyses were used. After calculating the crude odds ratios (OR) (Model 1), values were adjusted for age and sex (Model 2). They were then further adjusted for other confounding factors including smoking, hypertension, diabetes, and sunlight exposure times (Model 3). All variables considered for the logistic regression analyses were examined for multicollinearity, and only variables with a variance inflation factor <5 were used. *P* values were two-tailed, and a *P* value < 0.05 indicated statistical significance.

## Results

Of the 17,186 eligible subjects aged >19 years who had their serum 25-hydroxyvitamin D levels measured, 16,396 (95.4%) subjects had their cataract statuses examined. The prevalence of DES is 9.8% (Standard error [SE], 0.5%). The prevalence of DES is significantly higher in women (mean: 15.0%, SE: 0.9%) than in men (mean: 4.5%, SE: 0.5%, *P* < 0.001). The demographic characteristics of these subjects according to their DES statuses are summarized in Table 1, while data standardized by age and sex are shown in S1 Table. Average serum 25-hydroxyvitamin D levels were 17.1 ng/mL, and the prevalence of DES was 9.8% (un-weighted number = 1,679). Subjects with DES were more likely to be female (*P* < 0.001), older (*P* = 0.009), and non-smokers (*P* < 0.001), and were more likely to have lower diastolic blood pressures (*P* < 0.001), lower triglycerides (*P* = 0.006), and shorter sun-exposure times (*P* = 0.005).

**Table 1:** Demographic and clinical characteristics, according to dry eye syndrome (DES) status, as reported in the Korean National Health and Nutrition Examination Survey 2010–2012.

Characteristics	DES (n = 1679)	No DES (n = 14717)	*p*	Participants (n = 16396)	no exam (n = 2383)	*p*	Total(n = 18779)
**Male (%)**	22.7 (2.1)	52.4 (0.7)	< .001*	49.5 (0.6)	47.8 (3.1)	.256	49.4 (0.5)
**Age (yrs)**	47.7 (1.0)	44.9 (0.3)	.009	46.3 (0.5)	44.0 (1.2)	.357	44.6
**Body mass index (kg/m^2^)**	23.4 (0.1)	23.7 (0.1)	.149	23.6 (0.1)	24.2 (0.2)	.054	23.9 (0.1)
**Systolic blood pressure (mmHg)**	117.1 (0.9)	119.1 (0.3)	.063	118.1 (0.4)	119.3 (1.4)	.752	119.1 (0.7)
**Diastolic blood pressure (mmHg)**	74.1 (0.5)	76.9 (0.2)	< .001*	75.5 (0.2)	76.6 (0.2)	.231	75.9 (0.5)
**Fasting glucose (mg/dL)**	96.4 (1.0)	96.8 (0.3)	.763	96.6 (0.5)	100.2 (2.1)	.120	98.5 (1.1)
**HbA1c (%)**	5.71 (0.1)	5.73 (0.0)	.714	5.72 (0.0)	5.89 (0.1)	.141	5.81 (0.0)
**Total cholesterol (mg/dL)**	187.7 (2.0)	187.9 (0.7)	.914	187.8 (1.0)	188.2 (3.6)	.926	188.0 (1.5)
**Triglyceride (mg/dL)**	122.7 (4.7)	136.8 (2.1)	.056	129.8 (2.6)	145.4 (12.4)	.202	137.4 (6.3)
**25-hydroxyvitamin D (ng/mL)**	16.9 (0.3)	17.4 (0.1)	.182	17.1 (0.2)	17.7 (0.4)	.426	17.5 (0.2)
**Diabetes (%)**	7.2 (1.4)	8.6 (0.6)	.376	8.5 (0.5)	11.9 (2.8)	.187	8.6 (0.5)
**Hypertension (%)**	31.9 (2.9)	30.4 (0.9)	.627	30.5 (0.9)	36.6 (4.3)	.106	30.7 (0.9)
**Sun exposure (%)**			.005			.732	
**< 2hrs/day**	68.9 (2.5)	60.0 (1.1)		60.9 (1.0)	62.0 (3.7)		60.9 (1.0)
**2–5 hrs/day**	22.7 (2.2)	26.8 (0.9)		26.5 (0.8)	29.1 (3.3)		26.5 (0.8)
**> 5hrs/day**	8.4 (1.8)	13.2 (0.8)		12.6 (0.8)	8.9 (2.0)		12.5 (0.7)
**Smoking status**			< .001*			.134	
**Never (%)**	75.2 (2.4)	53.4 (0.8)		55.6 (0.7)	48.2 (4.4)		55.3 (0.7)
**Former (%)**	14.9 (2.0)	20.9 (0.7)		20.3 (0.7)	19.9 (2.9)		20.3 (0.6)
**Current (%)**	9.9 (1.6)	25.7 (0.7)		24.1 (0.7)	31.8 (4.0)		24.4 (0.7)

Data are expressed as weighted means or weighted frequency (%) with standard errors.

* p < 0.05

The demographic and clinical characteristics of the patients according to blood 25-hydroxyvitamin D quintiles showed that as serum 25-hydroxyvitamin D levels increased, participants were more likely to be male (*P* trend < 0.001), older (*P* trend < 0.001), hypertensive (*P* trend = 0.001), and smokers (*P* trend < 0.001), and were more likely to have higher total cholesterol (*P* trend = 0.010) and have experienced longer sun exposures (*P* < 0.001, Table 2, and S2 Table after standardization by age and sex). Average blood 25-hydroxyvitamin D levels were 17.3 ng/mL (95% confidence interval [CI], 17.0–17.6). Men had significantly higher blood 25-hydroxyvitamin D levels (18.3 ng/mL, 95% CI, 17.9–18.6) than women (16.4 ng/mL, 95% CI, 16.0–16.8, *P* < 0.001). Women, but not men, showed significant differences in their serum 25-hydroxyvitamin D levels in relation to the presence of hypertension (*P* < 0.001, Table 3). The prevalence of vitamin D deficiency is 71.6 ± 1.1%, and the prevalence of vitamin D deficiency was higher in women (65.8 ± 1.4%) than in men (77.3 ± 1.3%, *P* < 0.001).

**Table 2:** Demographic and clinical characteristics by quartile blood 25-Hydroxyvitamin D categories among representative Korean adults aged 19 years or older.

Characteristics		Quartile blood 25-Hydroxyvitamin D level (ng/mL)				
	< 12.3	12.3–15.2	15.2–18.0	18.0–21.9	> 21.9	P for trend
**Unweighted number**	3280	3286	3272	3285	3273	
**Male (%)**	37.7 (1.6)	43.2 (1.8)	49.3 (1.7)	59.7 (1.9)	58.5 (2.0)	< .001*
**Age (yrs)**	41.2 (0.7)	42.8 (0.6)	45.2 (0.6)	47.1 (0.6)	50.3 (0.6)	< .001*
**Body mass index (kg/m**^**2**^**)**	23.2 (0.1)	23.8 (0.1)	23.8 (0.1)	24.1 (0.1)	23.6 (0.1)	.043
**Systolic blood pressure (mmHg)**	117.2 (0.6)	117.0 (0.5)	117.8 (0.6)	120.4 (0.7)	121.8 (0.7)	< .001*
**Diastolic blood pressure (mmHg)**	75.7 (0.4)	76.1 (0.4)	76.1 (0.3)	77.7 (0.4)	77.5 (0.4)	.001*
**Fasting glucose (mg/dL)**	95.7 (0.9)	96.6 (0.8)	96.7 (0.8)	97.2 (0.7)	97.6 (0.7)	.497
**HbA1c (%)**	5.6 (0.0)	5.7 (0.0)	5.7 (0.0)	5.7 (0.0)	5.8 (0.0)	.240
**Total cholesterol (mg/dL)**	184.7 (1.6)	187.4 (1.3)	186.3 (1.4)	191.6 (1.3)	189.7 (1.6)	.010*
**Triglyceride (mg/dL)**	132.6 (4.6)	138.6 (6.2)	129.9 (3.4)	144.9 (4.2)	131.8 (3.6)	.046
**Diabetes (%)**	8.6 (1.4)	8.2 (1.1)	8.7 (1.1)	7.6 (1.1)	9.3 (1.1)	.869
**Hypertension (%)**	27.7 (1.9)	25.9 (1.7)	30.4 (2.0)	31.5 (2.1)	36.9 (2.0)	.001*
**Sun exposure (>5hrs/day, %)**						< .001*
** < 2hrs/day**	68.8 (1.8)	66.6 (1.8)	63.6 (2.0)	55.6 (2.0)	49.3 (2.3)	
**2–5 hrs/day**	24.2 (1.7)	24.8 (1.6)	25.1 (1.7)	29.9 (1.8)	28/3 (1.8)	
** > 5hrs/day**	7.0 (1.1)	8.6 (1.2)	11.4 (1.4)	14.5 (1.5)	12.7 (0.8)	
**Smoking status**						< .001*
**Never (%)**	61.4 (1.9)	60.3 (1.8)	55.9 (1.9)	50.9 (2.0)	49.2 (1.9)	
**Former (%)**	16.4 (1.4)	16.5 (1.4)	20.0 (1.6)	23.7 (1.7)	25.2 (1.6)	
**Current(%)**	22.3 (1.6)	23.2 (1.7)	24.1 (1.6)	25.4 (1.7)	25.7 (1.7)	

Data are expressed as weighted means or weighted frequency (%) with standard errors.

* p < 0.05

**Table 3:** Gender difference of blood 25-Hydroxyvitamin D levels (ng/mL) of men and women according to age group and other variables among representative Korean adults aged 19 years or older. Blood 25-hydroxyvitamin D levels were expressed as weighted estimates [%] (standard errors [%], 95% confidence intervals).

Characteristics	Blood 25-Hydroxyvitamin D level (ng/mL)			
	Men (n = 7080)	P	Women (n = 9316)	P
**All subjects aged 19+ years**				
**Age groups**		< .001*		< .001*
19–29 yrs	16.5 (0.2)		14.4 (0.2)	
30–39 yrs	17.4 (0.3)		15.9 (0.2)	
40–49 yrs	18.7 (0.3)		16.0 (0.2)	
50–59 yrs	19.6 (0.2)		17.3 (0.2)	
60–69 yrs	20.2 (0.3)		18.4 (0.3)	
70+	18.8 (0.6)		18.1 (0.3)	
**Dry eye syndrome**		.953		.807
None	18.2 (0.6)		16.5 (0.4)	
Dry eye syndrome	18.3 (0.1)		16.4 (0.2)	
**Hypertension**		.217		< .001*
Non-hypertension	18.2 (0.2)		16.1 (0.1)	
Hypertension	18.6 (0.2)		17.4 (0.3)	
**Sun exposure**		< .001*		< .001*
< 2hrs/day	17.6 (0.2)		15.9 (0.2)	
2–5 hrs/day	18.3 (0.3)		16.8 (0.3)	
> 5hrs/day	20.1 (0.4)		19.1 (0.7)	
**Smoking status**		.011		< .001*
Never	17.8 (0.3)		16.6 (0.2)	
Former	18.7 (0.2)		15.3 (0.4)	
Current	18.0 (0.2)		14.8 (0.4)	

Data are expressed as weighted means or weighted frequency (%) with standard errors.

* p < 0.05

**Fig 2:**
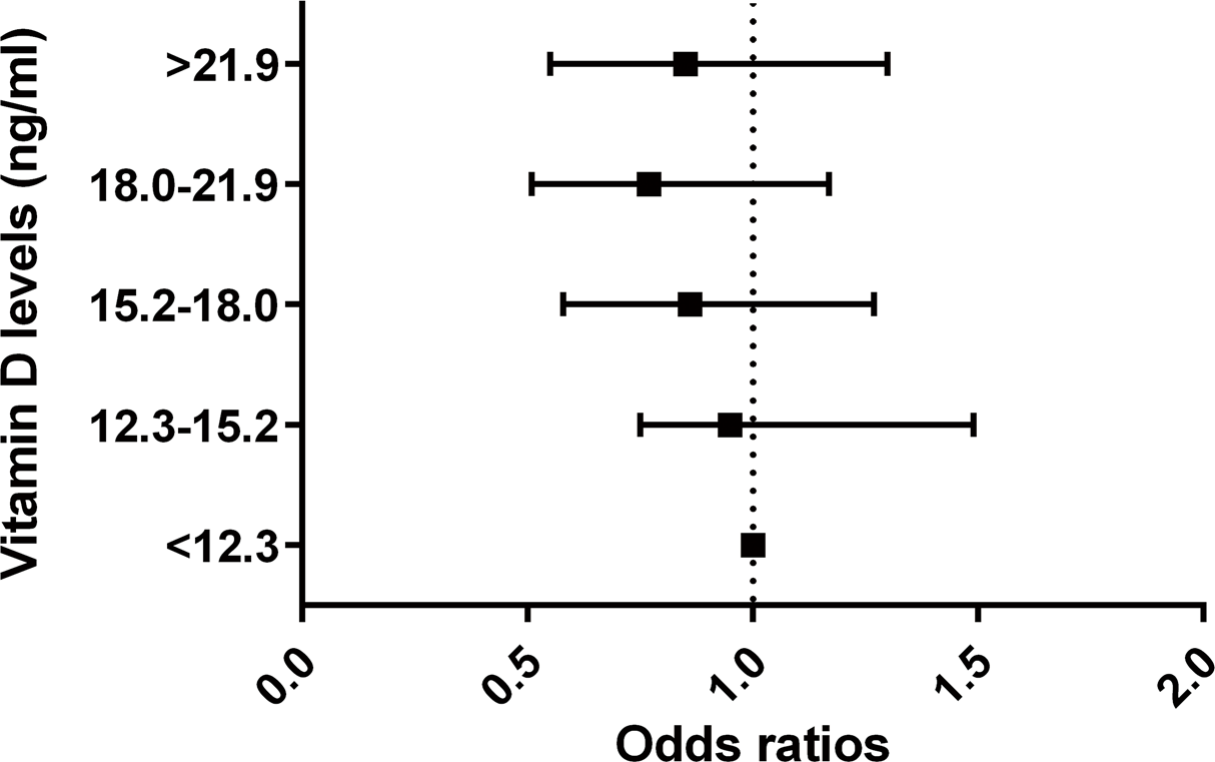
The odds ratios of dry eye syndrome according to quintiles of blood vitamin D levels (reference group = <12.3 ng/ml)

The odds of DES significantly decreased across the quintiles of the serum 25-hydroxyvitamin D levels (P for trend = 0.026, Model 1). However, this association was attenuated after adjusting for potential confounders including age, sex, hypertension, diabetes, smoking, and sun light exposure times (P for trend = 0.076, Model 3). Higher serum 25-hydroxyvitamin D levels were significantly associated with the decreasing odds of DES (Model1, quintile 5 versus 1, OR = 0.73, 95%CI: 0.51–0.97). However, this association was also attenuated after adjusting for potential confounders (Model 3, quintile 5 versus 1, OR = 0.85, 95%CI: 0.55–1.30, Fig 2). In subgroup analysis by gender, the OR of DES in those with higher serum 25-hydroxyvitamin D levels versus those with low levels was lower in men than women (Model 3, quintile 5 versus 1, adjusted OR = 0.70 in men, OR = 0.92 in women), but this was not statistically significant (Table 4). The prevalence of DES was not different between subjects with and without vitamin D deficiency (Table 5). The relative odds of DR (OR = 0.37, 95% CI: 0.18–0.76)[15] and late-AMD (OR = 0.32, 95% CI: 0.12–0.81)[14] in subjects with the highest vitamin D quintile versus the lowest one were relatively low, whereas the relative odds of cataract (OR = 0.76, 95% CI: 0.59–0.99)[16] and DES were relatively high in men (Table 6). In women, serum vitamin D levels were not associated with ocular diseases.

**Table 4:** Association between blood 25-hydroxyvitamin D and prevalence of dry eye syndrome (DES) among representative Korean adults.

Vitamin D quintiles (ng/mL)	Case/total number	Prevalence	Model 1	Model 2	Model 3
**Both gender**		**9.8 (0.5)**			
Quintile 1 (<12.34)	384/2896	10.6 (1.3)	1.00 (reference)	1.00 (reference)	1.00 (reference)
Quintile 2 (12.34–15.27)	382/2904	10.3 (1.2)	0.97 (0.74–1.43)	0.93 (0.71–1.41)	0.95 (0.75–1.49)
Quintile 3 (15.27–18.03)	315/2957	9.7 (1.1)	0.90 (0.63–1.28)	0.85 (0.59–1.22)	0.86 (0.58–1.27)
Quintile 4 (18.03–21.98)	305/2980	8.9 (1.2)	0.80 (0.54–1.18)	0.74 (0.50–1.10)	0.77 (0.51–1.17)
Quintile 5 (>21.98)	293/2980	8.5 (1.1)	0.73 (0.51–0.97)	0.71 (0.48–1.04)	0.85 (0.55–1.30)
P for trend		.043	.026	.025	.076
**Men**		**4.5 (0.5)**			
Quintile 1 (<13.47)	90/1327	5.1 (1.1)	1.00 (reference)	1.00 (reference)	1.00 (reference)
Quintile 2 (13.47–16.54)	67/1423	3.4 (0.9)	0.65 (0.33–1.27)	0.64 (0.33–1.26)	0.55 (0.25–1.19)
Quintile 3 (16.54–19.39)	81/1330	5.0 (1.2)	0.98 (0.50–1.91)	0.92 (0.48–1.76)	0.87 (0.44–1.73)
Quintile 4 (19.39–23.20)	84/1331	5.2 (1.1)	1.01 (0.55–1.87)	0.94 (0.50–1.78)	0.67 (0.31–1.47)
Quintile 5 (>23.20)	62/1352	3.9 (1.2)	0.76 (0.35–1.61)	0.68 (0.32–1.43)	0.70 (0.30–1.64)
P for trend		.690	.884	.625	.543
**Women**		**15.0 (0.9)**			
Quintile 1 (<11.64)	268/1867	14.3 (2.1)	1.00 (reference)	1.00 (reference)	1.00 (reference)
Quintile 2 (11.64–14.40)	280/1867	16.4 (1.9)	1.17 (0.76–1.80)	1.15 (0.75–1.78)	1.28 (0.81–2.01)
Quintile 3 (14.40–17.10)	256/1861	16.2 (2.0)	1.15 (0.74–1.80)	1.11 (0.71–1.75)	1.11 (0.68–1.79)
Quintile 4 (17.10–20.90)	245/1859	13.8 (2.1)	0.95 (0.60–1.50)	0.90 (0.57–1.42)	0.95 (0.58–1.55)
Quintile 5 (>20.90)	246/1862	14.2 (2.0)	0.99 (0.61–1.60)	0.90 (0.55–1.48)	0.92 (0.55–1.54)
P for trend		.841	.656	.386	.387

Prevalence was expressed as weighted estimates [%] (standard errors [%], 95% confidence intervals).

Model 1: Crude. Model 2: adjusted for sex and age. Model 3: adjusted for sex, age, diabetes, hypertension, sunlight exposure time, smoking, and body mass index.

**Table 5:** Prevalence of dry eye syndrome (DES) in subjects with or without vitamin D deficiency (25-hydroxyvitamin D < 20 ng/mL).

Prevalence of DES (%)	No Vitamin D deficiency	Vitamin D deficiency	*p*
**Both**	8.5 (0.9)	10.3 (0.6)	.123
**Men**	4.3 (0.8)	4.6 (0.6)	.720
**Women**	14.8 (2.0)	15.1 (1.0)	.902

Data are expressed as weighted means or weighted frequency (%) with standard errors.

**Table 6:** Relative association between serum 25-hydroxyvitamin D and ocular diseases including diabetic retinopathy (DR), vision threatening diabetic retinopathy (VTDR), age-related macular degeneration (AMD), cataract and dry eye syndrome (DES) among representative Korean adults.

Adjusted odds ratio of disease (quintile 5 versus 1)	Both gender	Men	Women
DR	0.66 (0.38–1.13)	0.37 (0.18–0.76)*	1.58 (0.78–3.20)
VTDR	0.64 (0.25–1.59)	0.30 (0.08–1.08)	1.97 (0.79–4.90)
Late AMD	0.75 (0.33–1.58)	0.32 (0.12–0.81)*	1.90 (0.66–5.44)
Cataract	0.86 (0.71–1.04)	0.76 (0.59–0.99)*	0.84 (0.66–1.07)
DES	0.85 (0.55–1.30)	0.70 (0.30–1.64)	0.92 (0.55–1.54)

* p < 0.05

## Discussion

The crude odds of DES significantly decreased across the quintiles of serum 25-hydroxyvitamin D levels, and higher serum 25-hydroxyvitamin D levels were significantly associated with decreasing odds of DES. However, these associations were attenuated after adjusting for confounders. In addition, the relative odds of DR and late AMD were relatively low, while those of cataract and DES were high.

After adjusting for potentially confounding factors including sex, age, smoking, hypertension, diabetes, and sunlight exposure times, subjects in the highest serum 25-hydroxyvitamin D quintile had a 15% lower risk of DES than subjects within the lowest quintile. However, the 95% CI of this association included the null point of 1.0 and did not show statistical significance. Our study is consistent with a previous cross sectional study that serum 25 vitamin D levels were not associated with the presence or severity of disease, although higher vitamin D levels were associated with decreased subjective DES symptoms [23]. However, other case-control studies reported that vitamin D deficiency was associated with the decreased tear break-up time and Shirmer test scores, which imply that vitamin D deficiency may be associated with dry eye symptoms [24, 25]. The present study did not find a significant inverse association between vitamin D and DES. We hypothesized that serum vitamin D has a limited effect on DES. Serum vitamin D may have difficulty in reaching the cornea, due to its lack of vasculature.

Several studies reported that vitamin D can be anti-inflammatory at the ocular surface. In a mouse model, topical administration of vitamin D inhibits migration of Langerhans cells and maturation, and delayed neovascularization of the central cornea [26]. In a rat keratoplasty model, topical vitamin D protected against keratoplasty rejection, inhibiting the pro-inflammatory cytokines interleukin-1 and tumor necrosis factor [27]. However, these studies used topical vitamin D and not serum vitamin D. We hypothesized that the effect of serum vitamin D on the cornea may be limited due to the decreased blood supply to the cornea.

**Fig 3:**
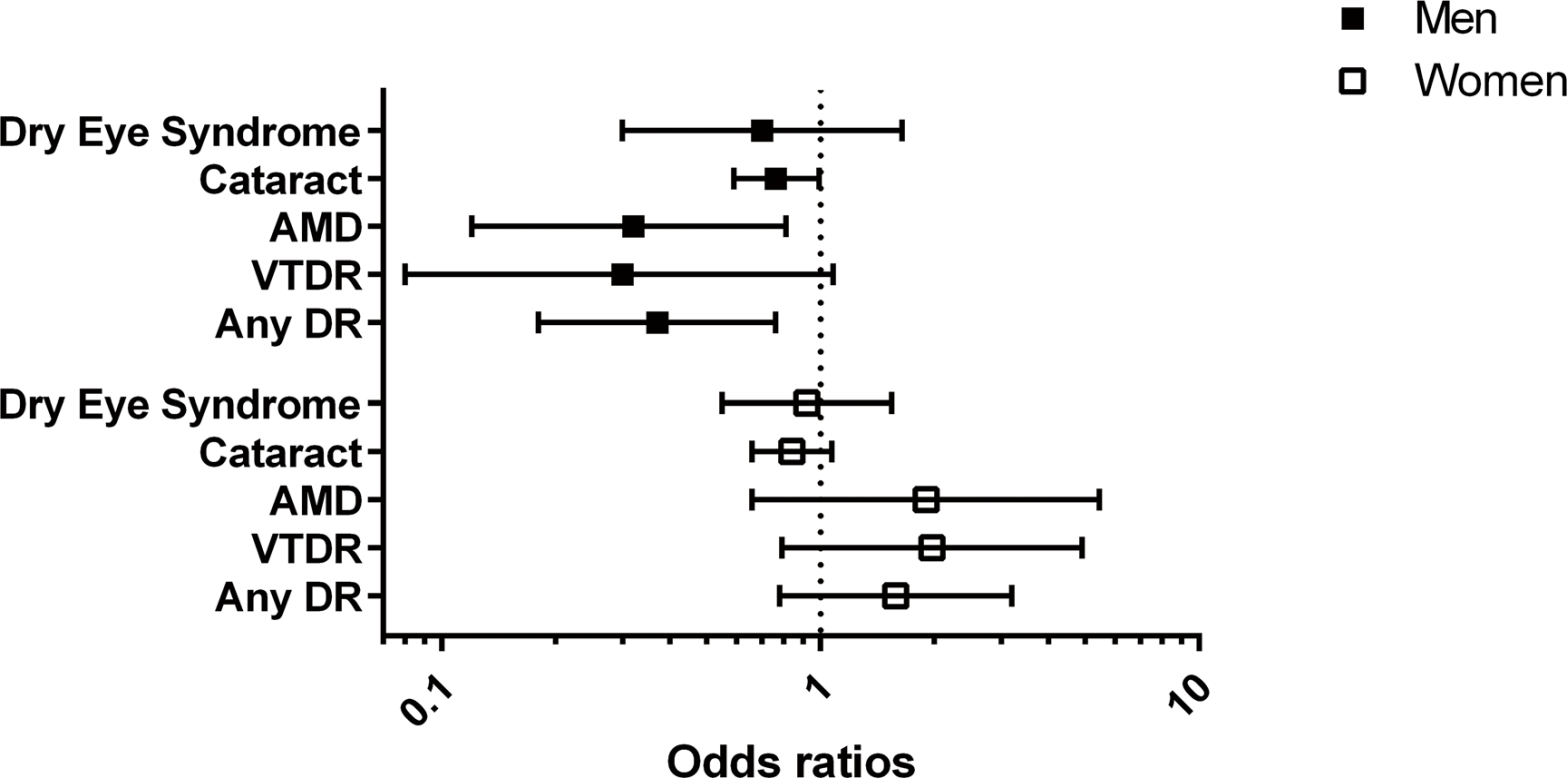
The comparison of odds ratios of dry eye syndrome (DES), cataract, age-related macular degeneration (AMD), any diabetic retinopathy (DR), and vision-threatening DR (VTDR) according to blood vitamin D levels (reference group = lowest vitamin D quintile group).

To further support our hypothesis, we compared the relative odds of DES in subjects in the highest vitamin D quintile versus those in the lowest quintile with their relative odds of other ocular diseases including DR, AMD, and cataract, from our previous studies using the same KNHANE population (Fig 3) [14–16]. In men, the relative odds of DR (OR = 0.37, 95% CI: 0.18–0.76) or late AMD (OR = 0.32, 95% CI: 0.12–0.81) are lower than cataract (OR = 0.76, 95% CI: 0.59–0.99) or DES (OR = 0.70, 95% CI: 0.30–1.64). The distribution of OR is consistent with the location of the lesion in ocular anatomy. The retina and macula, where relative lower ORs were observed, are located in the posterior part of the eye, whereas the lens and cornea, where relatively higher ORs were observed, are in the anterior part of the eye. The retina and macula have more blood supply than the lens and cornea do. The effect of serum vitamin D may be attenuated in case of decreased blood supply, because serum vitamin D reaches the eye through the blood supply. In addition, the lens may be more affected by sunlight (UVB), which is a risk factor for cataracts, than it is by vitamin D. On the contrary, the macula would be less influenced by the harmful effect of UVB, because only a small portion of UVB reaches to the retina. This suggests that different locations of ocular lesions may be the cause of the differential effects of vitamin D.

Another interesting finding is that the inverse association between vitamin D and ocular disease is significant in men, but not in women. Although the adjusted ORs of DR, AMD, and cataract in the highest quintile versus the lowest one were significantly decreased in men, such an association was not shown in women (Fig 3). Moreover, the ORs of DR and AMD in women have the opposite direction to those in men. One possible reason is that men are usually involved in more activities outside, which leads to greater exposure to sunlight than women. Sunlight exposure is the main driver for vitamin D production in the skin. Another possible reason is that women have reduced sensitivities to vitamin D, or they may more frequently use sunlight protective instruments such as clothes, sun block cream, hats, or sunglasses. However, this is purely speculative. Further studies are required to identify the factors responsible for this gender difference and to elucidate the exact sex-specific biological mechanisms.

The average vitamin D concentrations were low (17.1 ng/mL), which is lower than the vitamin D deficiency criteria (20.0 ng/mL). Moreover, vitamin D levels increased with age. These findings are supported by previous studies in which the prevalence of vitamin D insufficiency was about 70% or higher, and elderly people had higher vitamin D levels compared with young people in countries such as Korea and Thailand [28–31]. One possible explanation is that aged persons have more opportunity to spend time to do outdoor activities. The rapid economic development in Korea often means that young people have indoor jobs, whereas old persons have outdoor jobs [29].

Alternative explanation for result of the present study is that DES may be associated with vitamin D receptor dysfunction, not serum 25-hydroxyvitamin D levels [32]. Unfortunately, we could not confirm it, because KNHANES did not have information on the vitamin D receptor dysfunction. In addition, it is possible that vitamin D may get direct access to the cornea. Vitamin D is a fat-soluble steroid that should be taken up by the lacrimal gland and possibly secreted in tears onto the ocular surface.

The results from this study warrant further investigation. Vitamin D levels differ according to the latitude, ethnicity, and culture of the study population. Vitamin D production in the skin varies with UVB exposure, which is affected by many factors including sun bathing, time spent outside, clothing traditions, the use of sun protection agents, and skin color [33]. In addition, exposure to UVB declines from the equator to the polar regions, creating a gradient of vitamin D production in the skin. Thus, the effect of vitamin D on ocular diseases may differ in different parts of the world, which needs further study in the future.

In the present study only serum 25-hydroxyvitamin D levels were examined to assess the vitamin D status. However, recent advancements in technology make the measurement of metabolites of vitamin D possible [34]. Given that measuring only 25-hydroxyvitamin D cannot give the complete explanation about association between vitamin D and disease, further study is needed to include the measuring vitamin D metabolites.

The relatively large number of participants and the study’s design are the major strengths of this study. Another strength is the rigorous quality control for the measurements of serum 25-hydroxyvitamin D levels. This study also has several limitations. First, DES was evaluated by a self-reported method that was not based on the clinical examination, which may cause bias in the diagnosis of DES. The questionnaire did not include the current status of DES and that of potential patients. Even though DES is a complex disorder, with subtypes such as tear deficiency DES and evaporative DES, the severity of DES was not assessed by measures such as the keratoconjunctival epithelial damage score in the present study. Second, it was not possible to adjust for seasonal variations in serum 25-hydroxyvitamin D levels because KNHANES does not contain information about the dates on which the blood samples were taken. A recent study showed that an Asian population did not display any significant seasonal variations in its vitamin D status [35], whereas, another study reported significant seasonal variation, with lower vitamin D levels in winter [36]. In addition, we could not control for potential confounders DES-like symptoms such as recurrent corneal erosion, conjunctivochalasiasis, various neuropathies, or epithelial basement membrane dystrophy, and DES-inducing conditions such as depression, rheumatoid arthritis, or contact lens wear, which were not available in KNHANES. Third, ocular vitamin D levels were not examined. Serum vitamin D levels may not reflect ocular vitamin D levels. Finally, the current study is cross-sectional in its design, which makes inferring causality difficult.

To the best of our knowledge, the study provides the first population-based epidemiologic data on the associations between serum 25-hydroxyvitamin D levels and DES. We did not find a significant inverse association between serum 25-hydroxyvitamin D levels and DES in a representative Korean population. For prevention or treatment of DES, topical vitamin D is likely to be better rather than systemic vitamin D. Furthermore, our study suggests a differential effect of vitamin D on DR, AMD, cataract, and DES. The effect of vitamin D on these ocular diseases may differ by the location of the lesion in the eye.

## Supporting Information

S1 Table Age- and Sex- standardized demographic and clinical characteristics. (DOC)

S2 Table Demographic characteristics by quartile blood 25-Hydroxyvitamin D categories. (DOC)

## References

[1] Wei Y, Asbell PA. The core mechanism of dry eye disease is inflammation. Eye & contact lens. 2014;40(4):248–56. Epub 2014/11/13. 10.1097/icl.0000000000000042 ; PubMed Central PMCID: PMCPmc4231828. 25390549 PMC4231828

[2] Uchino Y, Kawakita T, Miyazawa M, Ishii T, Onouchi H, Yasuda K, et al Oxidative stress induced inflammation initiates functional decline of tear production. PLoS One. 2012;7(10):e45805 Epub 2012/10/17. 10.1371/journal.pone.0045805 ; PubMed Central PMCID: PMCPmc3465290. 23071526 PMC3465290

[3] Wakamatsu TH, Dogru M, Tsubota K. Tearful relations: oxidative stress, inflammation and eye diseases. Arquivos brasileiros de oftalmologia. 2008;71(6 Suppl):72–9. Epub 2009/03/11. . 19274416 10.1590/s0004-27492008000700015

[4] Jee D, Park SH, Kim MS, Kim EC. Antioxidant and inflammatory cytokine in tears of patients with dry eye syndrome treated with preservative-free versus preserved eye drops. Invest Ophthalmol Vis Sci. 2014;55(8):5081–9. Epub 2014/07/06. 10.1167/iovs.14-14483 . 24994869

[5] Jee D, Park M, Lee HJ, Kim MS, Kim EC. Comparison of treatment with preservative-free versus preserved sodium hyaluronate 0.1% and fluorometholone 0.1% eyedrops after cataract surgery in patients with preexisting dry-eye syndrome. Journal of cataract and refractive surgery. 2014 Epub 2014/12/10. 10.1016/j.jcrs.2014.11.034 . 25487027

[6] Alvarez JA, Chowdhury R, Jones DP, Martin GS, Brigham KL, Binongo JN, et al Vitamin D status is independently associated with plasma glutathione and cysteine thiol/disulfide redox status in adults. Clinical endocrinology. 2014 Epub 2014/03/19. 10.1111/cen.12449 . 24628365 PMC4115025

[7] Uberti F, Lattuada D, Morsanuto V, Nava U, Bolis G, Vacca G, et al Vitamin D protects Human Endothelial Cells from oxidative stress through the autophagic and survival pathways. The Journal of clinical endocrinology and metabolism. 2013:jc20132103. Epub 2013/11/29. 10.1210/jc.2013-2103 . 24285680

[8] Mangge H, Weghuber D, Prassl R, Haara A, Schnedl W, Postolache TT, et al The Role of Vitamin D in Atherosclerosis Inflammation Revisited: More a Bystander than a Player? Current vascular pharmacology. 2013 Epub 2013/12/18. . 24329737 10.2174/1570161111666131209125454

[9] Grant W, Strange R, Garland C. Sunshine is good medicine. The health benefits of ultraviolet-B induced vitamin D production. Journal of cosmetic dermatology. 2003;2(2):86–98. 17156062 10.1111/j.1473-2130.2004.00041.x

[10] Choi JA, Han K, Park YM, La TY. Low Serum 25-Hydroxyvitamin D Is Associated with Myopia in Korean Adolescents. Invest Ophthalmol Vis Sci. 2014 Epub 2014/02/01. 10.1167/iovs.13-12853 . 24699557

[11] Millen AE, Voland R, Sondel SA, Parekh N, Horst RL, Wallace RB, et al Vitamin D status and early age-related macular degeneration in postmenopausal women. Archives of ophthalmology. 2011;129(4):481 10.1001/archophthalmol.2011.48 21482873 PMC3075411

[12] Payne JF, Ray R, Watson DG, Delille C, Rimler E, Cleveland J, et al Vitamin D insufficiency in diabetic retinopathy. Endocrine Practice. 2012;18(2):185–93. 10.4158/EP11147.OR 21940279 PMC4706181

[13] Patrick PA, Visintainer PF, Shi Q, Weiss IA, Brand DA. Vitamin D and retinopathy in adults with diabetes mellitus. Arch Ophthalmol. 2012;130(6):756–60. Epub 2012/07/18. 10.1001/archophthalmol.2011.2749 . 22801837

[14] Kim EC, Han K, Jee D. Inverse Relationship between High Blood 25-Hydroxyvitamin D and Late Stage of Age-Related Macular Degeneration in a Representative Korean Population. Invest Ophthalmol Vis Sci. 2014 Epub 2014/07/13. 10.1167/iovs.14-14763 . 25015360

[15] Jee D, Han K, Kim EC. Inverse association between high blood 25-hydroxyvitamin D levels and diabetic retinopathy in a representative Korean population. PLoS One. 2014;9(12):e115199 Epub 2014/12/09. 10.1371/journal.pone.0115199 ; PubMed Central PMCID: PMCPmc4259490. 25485770 PMC4259490

[16] Jee D, Kim EC. Association between serum 25-hydroxyvitamin D levels and age-related cataracts. Journal of cataract and refractive surgery. 2015;41(8):1705–15. Epub 2015/10/04. 10.1016/j.jcrs.2014.12.052 . 26432129

[17] Yin Z, Pintea V, Lin Y, Hammock BD, Watsky MA. Vitamin D Enhances Corneal Epithelial Barrier Function. Investigative Ophthalmology & Visual Science. 2011;52(10):7359–64. 10.1167/iovs.11-7605 . 21715350 PMC3183972

[18] Kim Y, Park S, Kim NS, Lee BK. Inappropriate survey design analysis of the Korean National Health and Nutrition Examination Survey may produce biased results. Journal of preventive medicine and public health = Yebang Uihakhoe chi. 2013;46(2):96–104. Epub 2013/04/11. 10.3961/jpmph.2013.46.2.96 ; PubMed Central PMCID: PMCPmc3615385. 23573374 PMC3615385

[19] Park HA. The Korea national health and nutrition examination survey as a primary data source. Korean journal of family medicine. 2013;34(2):79 Epub 2013/04/06. 10.4082/kjfm.2013.34.2.79 ; PubMed Central PMCID: PMCPmc3611106. 23560205 PMC3611106

[20] Sempos CT, Vesper HW, Phinney KW, Thienpont LM, Coates PM. Vitamin D status as an international issue: national surveys and the problem of standardization. Scandinavian journal of clinical and laboratory investigation Supplementum. 2012;243:32–40. Epub 2012/06/08. 10.3109/00365513.2012.681935 . 22536760

[21] Ahn JM, Lee SH, Rim TH, Park RJ, Yang HS, Kim TI, et al Prevalence of and risk factors associated with dry eye: the Korea National Health and Nutrition Examination Survey 2010–2011. Am J Ophthalmol. 2014;158(6):1205–14.e7. Epub 2014/08/26. 10.1016/j.ajo.2014.08.021 . 25149910

[22] Chun YH, Kim HR, Han K, Park YG, Song HJ, Na KS. Total cholesterol and lipoprotein composition are associated with dry eye disease in Korean women. Lipids in health and disease. 2013;12:84 Epub 2013/06/06. 10.1186/1476-511x-12-84 ; PubMed Central PMCID: PMCPmc3680171. 23734839 PMC3680171

[23] Galor A, Gardener H, Pouyeh B, Feuer W, Florez H. Effect of a Mediterranean dietary pattern and vitamin D levels on Dry Eye syndrome. Cornea. 2014;33(5):437–41. Epub 2014/03/14. 10.1097/ico.0000000000000089 ; PubMed Central PMCID: PMCPmc3972326. 24622300 PMC3972326

[24] Yildirim P, Garip Y, Karci AA, Guler T. Dry eye in vitamin D deficiency: more than an incidental association. International journal of rheumatic diseases. 2015 Epub 2015/08/14. 10.1111/1756-185x.12727 . 26269110

[25] Kurtul BE, Ozer PA, Aydinli MS. The association of vitamin D deficiency with tear break-up time and Schirmer testing in non-Sjogren dry eye. Eye (London, England). 2015;29(8):1081–4. Epub 2015/06/13. 10.1038/eye.2015.96 ; PubMed Central PMCID: PMCPmc4541362. 26066054 PMC4541362

[26] Suzuki T, Sano Y, Kinoshita S. Effects of 1alpha,25-dihydroxyvitamin D3 on Langerhans cell migration and corneal neovascularization in mice. Invest Ophthalmol Vis Sci. 2000;41(1):154–8. Epub 2000/01/14. . 10634615

[27] Dang ST, Lu XH, Zhou J, Bai L. [Effects of 1alpha, 25-dihydroxyvitamin D3 on the acute immune rejection and corneal neovascularization in high-risk penetrating keratoplasty in rats]. Di 1 jun yi da xue xue bao = Academic journal of the first medical college of PLA. 2004;24(8):892–6, 903 Epub 2004/08/24. . 15321754

[28] Nimitphong H, Holick MF. Vitamin D status and sun exposure in southeast Asia. Dermato-endocrinology. 2013;5(1):34–7. Epub 2014/02/05. 10.4161/derm.24054 ; PubMed Central PMCID: PMCPmc3897596. 24494040 PMC3897596

[29] Choi HS, Oh HJ, Choi H, Choi WH, Kim JG, Kim KM, et al Vitamin D insufficiency in Korea—a greater threat to younger generation: the Korea National Health and Nutrition Examination Survey (KNHANES) 2008. The Journal of clinical endocrinology and metabolism. 2011;96(3):643–51. Epub 2010/12/31. 10.1210/jc.2010-2133 . 21190984

[30] Chailurkit LO, Aekplakorn W, Ongphiphadhanakul B. Regional variation and determinants of vitamin D status in sunshine-abundant Thailand. BMC public health. 2011;11:853 Epub 2011/11/15. 10.1186/1471-2458-11-853 ; PubMed Central PMCID: PMCPmc3247919. 22074319 PMC3247919

[31] Lu L, Yu Z, Pan A, Hu FB, Franco OH, Li H, et al Plasma 25-hydroxyvitamin D concentration and metabolic syndrome among middle-aged and elderly Chinese individuals. Diabetes Care. 2009;32(7):1278–83. Epub 2009/04/16. 10.2337/dc09-0209 ; PubMed Central PMCID: PMCPmc2699709. 19366976 PMC2699709

[32] Waterhouse JC, Perez TH, Albert PJ. Reversing bacteria-induced vitamin D receptor dysfunction is key to autoimmune disease. Annals of the New York Academy of Sciences. 2009;1173:757–65. Epub 2009/09/18. 10.1111/j.1749-6632.2009.04637.x . 19758226

[33] Hagenau T, Vest R, Gissel T, Poulsen C, Erlandsen M, Mosekilde L, et al Global vitamin D levels in relation to age, gender, skin pigmentation and latitude: an ecologic meta-regression analysis. Osteoporosis international. 2009;20(1):133–40. 10.1007/s00198-008-0626-y 18458986

[34] Muller MJ, Volmer DA. Mass spectrometric profiling of vitamin D metabolites beyond 25-hydroxyvitamin D. Clinical chemistry. 2015;61(8):1033–48. Epub 2015/07/02. 10.1373/clinchem.2015.241430 . 26130585

[35] Smith M. Seasonal, ethnic and gender variations in serum vitamin D3 levels in the local population of Peterborough. Bioscience Horizons. 2010;3(2):124–31.

[36] Lee YA, Kim HY, Hong H, Kim JY, Kwon HJ, Shin CH, et al Risk factors for low vitamin D status in Korean adolescents: the Korea National Health and Nutrition Examination Survey (KNHANES) 2008–2009. Public health nutrition. 2014;17(4):764–71. Epub 2013/03/07. 10.1017/s1368980013000438 . 23462341 PMC10282207

