# Supplementary material for: Serum 25-Hydroxyvitamin D Levels and Dry Eye Syndrome: Differential Effects of Vitamin D on Ocular Diseases
Source: PLoS One. 2016 Feb 19;11(2):e0149294. doi: 10.1371/journal.pone.0149294 (PMC4760949; doi:10.1371/journal.pone.0149294)
Supplement: S1 Table — (DOC) [file pone.0149294.s001.doc]

**S1 Table.** Age- and Sex- standardized demographic and clinical characteristics, according to dry eye syndrome (DES) status, as reported in the Korean National Health and Nutrition Examination Survey 2010-2012

| **Characteristics** | **DES**  **(n =1679)** | **No DES**  **(n =14717)** | ***p*** | **Participants**  **(n =16396)** | **no exam**  **(n =2383)** | ***p*** | **Total**  **(n = 18779)** |
| --- | --- | --- | --- | --- | --- | --- | --- |
| **Male (%)** | N/A | N/A | N/A | N/A | N/A | N/A | N/A |
| **Age (yrs)** | N/A | N/A | N/A | N/A | N/A | N/A | N/A |
| **Body mass index (kg/m^2^)** | 23.5 (0.16) | 23.6 (0.05) | .112 | 23.6 (0.05) | 23.4 (0.16) | .420 | 23.6 (0.05) |
| **Systolic blood pressure (mmHg)** | 113.3 (0.57) | 115.4 (0.19) | .002 | 115.3 (0.18) | 115.9 (0.62) | .364 | 115.3 (0.18) |
| **Diastolic blood pressure (mmHg)** | 75.1 (0.40) | 76.3 (0.16) | .007 | 76.3 (0.15) | 76.3 (0.42) | .901 | 76.3 (0.15) |
| **Fasting glucose (mg/dL)** | 94.0 (0.70) | 94.6 (0.24) | .413 | 94.5 (0.23) | 98.5 (1.20) | .002 | 94.7 (0.23) |
| **HbA1c (%)** | 5.58 (0.03) | 5.63 (0.01) | .030 | 5.62 (0.01) | 5.83 (0.07) | .005 | 5.63 (0.01) |
| **Total cholesterol (mg/dL)** | 186.5 (1.36) | 186.7 (0.47) | .538 | 186.6 (0.47) | 184.4 (1.91) | .374 | 186.5 (0.46) |
| **Triglyceride (mg/dL)** | 126.1 (4.05) | 127.8 (1.33) | .330 | 127.5 (1.26) | 130.6 (5.29) | .626 | 127.7 (1.24) |
| **25-hydroxyvitamin D (ng/mL)** | 16.3 (0.26) | 16.8 (0.14) | .091 | 16.8 (0.13) | 17.7 (0.36) | .018 | 16.8 (0.13) |
| **Diabetes (%)** | 4.7 (0.86) | 5.6 (0.27) | .140 | 5.5 (0.26) | 7.8 (1.44) | .092 | 5.6 (0.25) |
| **Hypertension (%)** | 14.2 (1.44) | 19.4 (0.50) | .004 | 19.1 (0.48) | 21.3 (1.72) | .089 | 19.3 (0.46) |
| **Sun exposure (%)** |  |  | .039 |  |  | .646 |  |
| **< 2hrs/day** | 70.2 (2.24) | 64.7 (0.78) |  | 65.0 (0.75) | 60.0 (2.40) |  | 64.5 (0.73) |
| **2-5 hrs/day** | 21.9 (2.08) | 25.2 (0.69) |  | 25.0 (0.66) | 24.5 (1.75) |  | 24.9 (0.64) |
| **> 5hrs/day** | 7.8 (1.37) | 10.0 (0.54) |  | 9.9 (0.52) | 9.3 (1.17) |  | 9.9 (0.48) |
| **Smoking status** |  |  | 0.56 |  |  | .344 |  |
| **Never (%)** | 52.0 (1.74) | 52.9 (0.53) |  | 53.0 (0.51) | 37.0 (1.83) |  | 51.3 (0.51) |
| **Former (%)** | 20.2 (1.81) | 18.5 (0.47) |  | 18.6 (0.45) | 13.1 (1.36) |  | 18.2 (0.43) |
| **Current (%)** | 22.7 (2.18) | 24.0 (0.53) |  | 23.9 (0.52) | 20.1 (1.54) |  | 23.6 (0.50) |

Data are expressed as weighted means or weighted frequency (%) with standard errors. * p < 0.05
