# Supplementary material for: Serum 25-Hydroxyvitamin D Levels and Dry Eye Syndrome: Differential Effects of Vitamin D on Ocular Diseases
Source: PLoS One. 2016 Feb 19;11(2):e0149294. doi: 10.1371/journal.pone.0149294 (PMC4760949; doi:10.1371/journal.pone.0149294)
Supplement: S2 Table — (DOC) [file pone.0149294.s002.doc]

**S2 Table.** Demographic and clinical characteristics by quartile blood 25-Hydroxyvitamin D categories among representative Korean adults aged 19 years or older.

| **Characteristics** |  | **Quartile blood 25-Hydroxyvitamin D level (ng/mL)** | | | | | |  |
| --- | --- | --- | --- | --- | --- | --- | --- | --- |
|  | **< 12.3** | | **12.3-15.2** | **15.2-18.0** | **18.0-21.9** | **> 21.9** | **P for trend** | |
| **Unweighted number** | 3280 | | 3286 | 3272 | 3285 | 3273 |  | |
| **Male (%)** | N/A | | N/A | N/A | N/A | N/A | N/A | |
| **Age (yrs)** | N/A | | N/A | N/A | N/A | N/A | N/A | |
| **Body mass index (kg/m^2^)** | 23.3 (0.1) | | 23.9 (0.1) | 23.7 (0.1) | 23.5 (0.1) | 23.4 (0.1) | .700 | |
| **Systolic blood pressure (mmHg)** | 115.8 (0.4) | | 115.4 (0.4) | 114.7 (0.3) | 115.2 (0.4) | 115.6 (0.4) | .509 | |
| **Diastolic blood pressure (mmHg)** | 76.4 (0.3) | | 76.4 (0.3) | 75.9 (0.2) | 76.7 (0.3) | 76.4 (0.3) | .802 | |
| **Fasting glucose (mg/dL)** | 94.9 (0.6) | | 95.2 (0.5) | 94.7 (0.5) | 94.6 (0.5) | 93.3 (0.4) | .023 | |
| **HbA1c (%)** | 5.66 (0.03) | | 5.65 (0.02) | 5.63 (0.03) | 5.62 (0.02) | 5.58 (0.02) | .047 | |
| **Total cholesterol (mg/dL)** | 185.1 (1.0) | | 187.4 (0.9) | 185.1 (0.9) | 190.0 (0.9) | 186.3 (1.0) | .331 | |
| **Triglyceride (mg/dL)** | 137.3 (3.0) | | 132.8 (3.2) | 126.1 (2.7) | 127.3 (2.7) | 113.0 (2.2) | <0.001 | |
| **Diabetes (%)** | 5.9 (0.7) | | 6.2 (0.6) | 6.1 (0.5) | 5.1 (0.5) | 4.7 (0.6) | .012 | |
| **Hypertension (%)** | 20.4 (1.1) | | 20.3 (1.1) | 17.8 (0.9) | 18.8 (1.0) | 18.9 (1.1) | .157 | |
| **Sun exposure (>5hrs/day, %)** |  | |  |  |  |  | <.001 | |
| **< 2hrs/day** | 68.1 (1.3) | | 68.8 (1.4) | 66.6 (1.3) | 64.6 (1.4) | 55.1 (1.9) |  | |
| **2-5 hrs/day** | 23.7 (1.2) | | 23.8 (1.3) | 24.3 (1.2) | 25.0 (1.2) | 29.7 (1.7) |  | |
| **> 5hrs/day** | 8.0 (0.9) | | 7.3 (0.9) | 8.9 (0.8) | 10.1 (1.0) | 15.2 (1.3) |  | |
| **Smoking status** |  | |  |  |  |  | .011 | |
| **Never (%)** | 52.0 (1.1) | | 53.2 (1.0) | 52.4 (1.1) | 54.0 (1.3) | 52.3 (1.6) |  | |
| **Former (%)** | 16.4 (1.0) | | 17.7 (0.9) | 19.7 (1.0) | 18.1 (0.9) | 21.4 (1.5) |  | |
| **Current(%)** | 28.0 (1.2) | | 24.2 (1.1) | 23.4 (1.1) | 22.9 (1.1) | 21.7 (1.2) |  | |

Data are expressed as weighted means or weighted frequency (%) with standard errors. * p < 0.05
